# Race and Mental Health Among Older Adults: Within- and Between-Group Comparisons

**DOI:** 10.1093/geroni/igaa056

**Published:** 2020-12-16

**Authors:** Robert Joseph Taylor

**Affiliations:** School of Social Work, University of Michigan, Ann Arbor

This special issue of *Innovation in Aging* is expressly devoted to research on aging and mental health within racial and ethnic minority populations (e.g., African Americans, Asian Americans, Latinx). The lack of quality research on mental health for older members of racial and ethnic population groups has been a serious impediment to amassing a solid understanding of aging processes and contextual factors that are consequential for mental well-being in later life. This gap in the literature on aging is long-standing and particularly problematic given projected increases in the numbers of older adults from racial and ethnic minority population groups. Knowledge of the factors associated with mental health and aging for racial and ethnic minority adults is imperative for ensuring the personal well-being of older adults, their families, and communities. Further, this information is critical for the education and training of mental health practitioners who work with older adults in various settings (e.g., outpatient clinics, hospitals, nursing homes) and the development of mental health interventions and services that are culturally informed and congruent with the life experiences of racial and ethnic older adults.

The collected articles in this issue include reviews of the research literature on mental health issues among diverse race and ethnic groups, as well as empirical studies of risk (discrimination) and protective factors (social support, hopefulness) that affect mental health status. Specific population groups include African Americans, Latinx, and Asian Americans, as well as subgroups within these larger pan-ethnic categories. Research on mental health issues focus principally on depressive symptoms and anxiety within the context of time-use activities, everyday discrimination, loneliness, and trajectories and change in depression over time. Taken together, this work represents a fitting collection of research and scholarship that aptly demonstrates the benefits of research that has an explicit focus on older racial and ethnic minority populations. Please note that due to space limitations, this special issue does not cover all of the relevant issues affecting older adults’ mental health such as research on cognitive aging and substance abuse.

## COVID-19 and Racial Justice: Context Matters

In March 2020 when final manuscripts were due for this special issue, we could not have imagined the innumerable ways that the world and our personal and professional lives would be changed by the coronavirus pandemic and coronavirus disease 2019 (COVID-19). The disproportionate impact of COVID-19 on older populations and racial and ethnic minority groups (specifically Black Americans and Latinx Americans) underscored their precarious position in terms of higher levels of exposure, elevated medical risks for worse disease outcomes, and disproportionate mortality rates (Chatters et al., 2020). COVID-19 has revealed how racism and ageism (Chatters et al., 2020) continue to shape attitudes, practice and policies concerning older adults and racial and ethnic minority groups (Khazan, 2020). Racism and ageism fuel rhetoric regarding the origins of coronavirus (i.e., China Flu), physical vulnerabilities to disease attributed to individual behaviors (e.g., underlying health conditions), and characterizations of COVID-19 as a problem affecting specific populations (i.e., older adults and racial and ethnic minorities). Tragically, persons who are both older and members of racial and ethnic minority groups, particularly Black and Latinx older adults, continue to suffer disproportionally from COVID-19 as compared to younger persons and Whites, as well as compared to older White adults (Chatters et al., 2020).

Against the backdrop of the coronavirus pandemic, instances of racial injustice and violence continued unabated (CBS News, 2020). The public killing of George Floyd on May 5, 2020 precipitated nation-wide and global calls for racial justice and the end of police violence against Black and Latinx communities in the United States. This national reckoning was foreshadowed by a long history of racial violence against African Americans, Latinx, and Native Americans. Floyd’s death and subsequent protests against anti-Black racism and racial violence have initiated a profound awakening to the reality of various forms of structural racism that exist within our institutions, laws, practice, and policies—as well as our professions and fields of study.

It is important to note that although gerontology is much more inclusive than other fields and has become more inclusive than in the past, it still has a long way to go. When I attended one of my first Gerontological Society of America conferences in San Diego in the mid 1980s, there were only two sessions that significantly addressed older African Americans. Since that time, both the presence of African American researchers and the number of sessions of quality research addressing the needs of this population have substantially increased.

Despite this improvement, there are still many significant problems confronting the field of gerontology. There are too few members of minority groups who are editors and members of editorial boards of gerontology journals. Several of the major gerontology journals do not have any African Americans on their editorial boards and the vast majority of gerontology journals have never had an African American editor. The inclusion of African American gerontologists in these positions can provide perspectives on the importance of studies on racial and ethnic disparities, as well as expertise in reviewing articles on this topic.

Efforts to diversify knowledge about aging populations are often stymied by events that occur during the review process. Research on older African Americans is often rejected due to the lack of a comparison group (i.e., of non-Hispanic Whites). This occurs at many levels including reviews of manuscript submissions for peer review journals, as well as applications for National Institutes of Health funding. Further, many reviewers fail to understand that the number of quality data sets based on representative probability samples of minority groups are extremely limited. Consequently, it is not unusual for minority researchers, especially African American, to utilize data sets that are 20 years old because they still remain the best available data for the topics being addressed.

## Imagining an Inclusive Gerontology

Critiques of the field of gerontology have repeatedly affirmed the need for theoretical and conceptual frameworks that reflect and include information about the life circumstances and experiences of racial and ethnic minority older adults, as well as methodological improvements to ensure robust and rigorous research efforts. Scholars in psychology, sociology, and ethnic studies, as well as gerontology have voiced these concerns for the past 50 years. Scholars in the field of gerontology such as James S. Jackson, Rose Gibson, Peggye Dilworth-Anderson, Fernando Torres-Gil, Jacquelyn Jackson, Ron Angel, and Inabel Lindsay have held that within-group variability in racial and ethnic populations exists and matters for the study of aging. Further, thorough and sustained examination of the histories and life experiences of older racial and ethnic minority older adults is necessary for building an inclusive understanding of aging within and across diverse population groups.

Acknowledging and examining within-group differences mitigate against “essentialist” perspectives and stereotypical views about what aging looks like for racial and ethnic groups. Studies that merely focus on comparisons of racial and ethnic older adults with a White, non-Latinx reference group (i.e., between-group comparisons) reveal little about either group beyond the existence of difference (Taylor & Chatters, 2020). Further, the designation of the non-Latinx White population as a reference group is not value-neutral and inherently regards White as the accepted “norm” for the phenomenon being examined, whereas racial and ethnic minority groups are regarded as deviating from this norm. An exclusive focus on between-group comparisons effectively decontextualizes the personal circumstances and life histories of racial and ethnic minority older adults, a situation that is not remedied by statistical controls for socioeconomic position and other factors. The late James S. Jackson’s work in establishing the National Survey of Black Americans and the National Survey of American Life were revolutionary in that they both focused on within-group diversity. As a consequence, these two surveys have produced an unparalleled wealth of information about aging among African Americans as well as Black Caribbean immigrants. In summary, research and scholarship concerning the importance of within-group variability underscore crucial theoretical, conceptual, and methodological issues and concerns that confront the field and practice of gerontology. These issues and concerns are addressed by the contributing authors to this issue of *Innovation in Aging*.

## Race and Mental Health Among Older Adults: Within- and Between-Group Comparisons

The theme of this issue of *Innovation in Aging* represents a major development in the continuing reassessment of scholarship and research concerning the mental health of racial and ethnic minority older adults. The articles presented here take up questions of within- and between-group comparisons as they relate to the development of scholarship and research on racial and ethnic older adults and mental health. These articles explicitly frame questions about how exploring within-group variability informs our understanding of mental health for older racial and ethnic adults and complement recent work exploring within- and between-group differences in psychiatric disorders among older Black adults (Taylor & Chatters, 2020).

Of particular importance, several of the articles center the question of within-group variability by critiquing the use of *pan-ethnic categories* such as Asian American, Latinx, and Black American and the resulting invisibility of constituent groups that differ in religion, language, historical experience, culture, and race. The disaggregation of pan-ethnic categories thus represents another opportunity to explore within-group variability in life experiences and circumstances, differences that are consequential for understanding mental health. Finally, the articles in this issue are framed by and reference several overarching and inter-related perspectives and themes concerning human development including: cumulative advantage and disadvantage, risk and protective factors in mental health, the importance of social context and processes, and intersectionality of social identities.

Essentially, the articles presented here pose the question: What would the study of the mental health status of racial and ethnic minority older adults look like if our research took the exploration of within- and between-group diversity seriously?

## Overview of Articles

The articles represented in this special issue of *Innovation in Aging* cover a range of topics and concerns in the area of mental health. Prominent among them is a focus on within-group variability as it pertains to pan-ethnic categories. Notably, the articles by Taylor and Chatters (2020) on older Black Americans, Kim and colleagues (2020) on Asian American older adults, and Jimenez and colleagues (2020) on older Latinos provide extensive reviews of current research on the mental health of older adults with a focus on both between-group as well as within racial/ethnic group differences. In addition, the articles by Lincoln (2020) on obesity and mental health and by Nguyen (2020) on religion and mental health are in-depth reviews on these topics.

Although the article by Taylor and Chatters (2020) was published in an earlier issue of *Innovation in Aging*, I discuss it here because of its relevance to this special issue. The review of available literature on diagnosed psychiatric disorders (e.g., depression, anxiety) among older Black adults in Taylor and Chatters (2020) documents the appalling lack of information on this important public health issue. This situation is especially disappointing given that, over the life course, older Black adults have experienced life events and circumstances that are established risk factors for poor mental health. The study revealed that racial discrimination and negative interactions with family members are risk factors for psychiatric disorders among African Americans, whereas family support is not protective for disorders. Older age is associated with lower rates of psychiatric disorders among African Americans as compared to non-Latino Whites. However, a significant proportion of older African Americans who do report disorders do not seek professional help. Finally, as evidence of the importance of exploring ethnicity, Black Caribbean men have high rates of depression, posttraumatic stress disorder and suicide attempts, a pattern that runs counter to prior work on race and gender and mental health. Study findings confirm the value of an explicit focus on within-group differences in psychiatric disorders among older Black adults as a means to better understand how social identities (e.g., gender, ethnicity) and social resources (e.g., social supports, networks, and relationships) are associated with mental disorders.

Kim and colleagues (2020) provide an overview of the mental health status of older Asian American adults. This work is timely in reflecting the demographic growth of Asian American older adults in the United States, as well as underscoring the significant and meaningful ethnic, linguistic, cultural, and religious diversity in the more than 20 groups comprising the Asian American pan-ethnic designation. This article underscores that, in addition to recognizing ethnic and cultural distinctiveness among Asian Americans, it is crucial to acknowledge relevant life conditions that impact their social circumstances (e.g., immigration experiences and status). Both perspectives are needed to accurately assess mental health status and for developing appropriate and culturally responsive interventions. The authors provide important information regarding conceptual and methodological challenges and gaps in research on mental health among older Asian Americans and strategies going forward to improve mental health status.

Jimenez and colleagues (2020) address two common assumptions concerning mental health of older Latinos. As is the case with other racial/ethnic groups, pan-ethnic categorizations fail to account for within-group diversity (racial, ethnic, and cultural), resulting in simplistic and inaccurate portrayals of older Latinos overall. Their article focuses on older adults from three subgroups of older Latinos, Mexicans, Puerto Ricans, and Cubans, and uses epidemiological and clinical and psychopathology information on aging to demonstrate the inherent heterogeneity of the older Latino population. As the authors argue, this is specifically critical with respect to mental health status. Further, the presumed protective features of foreign nativity (i.e., immigrant paradox), whereby Latinos experience better mental health status despite greater social risks and less favorable social conditions, are questioned when considered within a life course perspective. Their article provides needed nuance for understanding issues of both diversity within the Latino category and how social inequalities over the life course impact the mental health of older Latinos.

In order to better understand and treat mental health issues of older Latinx groups, Mendez and colleagues (2020) focus on older Latinx adults’ attributions of mental health problems. They examine perceptions of mental health symptoms among older Mexican-origin Latinx and non-Latinx adult outpatient clients of a rural psychiatric clinic. Latent class analysis derived profiles of attributional beliefs and associated contextual factors indicated that older Mexican-origin Latinx and non-Latinx adults show both within- and between-group differences in attributions for mental health symptoms. Older Mexican-origin Latinx endorsed a unique Spiritual class (attributions to spiritual and/or supernatural causes), with higher endorsement from women. Both Mexican-origin Latinx and non-Latinx identified a Low Attribution class (mental health symptoms were not attributable to the causes listed). This work contributes to research verifying within-group heterogeneity in attributions of mental health symptoms among Latinx adults and the influences of demographic and contextual factors in these profiles.

Two articles that review current literature on mental health among older racial minority adults identify the lack of information and remaining questions that need to be addressed. Lincoln (2020) reviews current information and literature concerning the mental health impacts of obesity and identifies risk factors, causal mechanisms, and methodological approaches that help clarify the equivocal nature of the literature among older adults. Concerns about obesity and its physical health consequences for adults are acknowledged. However, mental health consequences of obesity are less understood among racial and ethnic minority older adults and, in several instances, findings are inconsistent with the general literature. Topic areas for future research are presented with an emphasis on the role of contextual factors, pathways, and mechanisms linking obesity and mental health, and more fully examining the role of population heterogeneity for understanding obesity and mental health connections across diverse groups.

Nguyen (2020) focuses on research on religion and mental health among older Black and Latino Americans. Religion has been important historically for these groups as a source of strength, and religious institutions have been a consistent resource of psychosocial support and tangible assistance and for building social capital for Black and Latino communities. Research on the relationship between the multiple dimensions of religiosity and mental health is discussed, including the conceptualization and measurement of both religion and mental health. Importantly, the review provides an understanding of religion as a multidimensional construct and how distinct dimensions of religion are associated with discrete mental indicators. Noted differences in religion–mental health relationships between Blacks, Latino, and non-Latino Whites are described. Directions for future research on religion and mental health in racial/ethnic minority populations, especially older minorities and practice implications and partnerships between clergy and mental health practitioners, are provided.

Several articles focus on depressive symptoms as experienced by older racial and ethnic minority adults. Byrd and colleagues’ (2020) research on the association between stress and depression explores the longitudinal and bidirectional relationships between depressive symptoms and perceived stress using data from the Baltimore Study of Black Aging—Patterns of Cognitive Aging. They find that depressive symptoms were associated with follow-up stress after accounting for baseline stress, chronic health conditions, and demographic factors (sex, education, and age), lending support to bidirectional relationships between psychosocial measures of stress and mental health for Black adults in later life. Further, this relationship was more pronounced for persons in their 60s versus those in their 50s, providing evidence of age differences in this association. Together their findings support ongoing efforts to better understand relationships between stress and mental health, specific features of the stress process, and possible age variations in how depression and stress are related.

Building on research and theory that exposure to objectively stressful events is associated with poorer mental health, Brown and colleagues (2020) examine the apparent paradox of older Black adults’ similar or better mental health status (anxiety, depressive symptoms) relative to Whites, despite clear race differences in cumulative exposures to stress. Using Health and Retirement Study (HRS) data, they examine Black–White differences in cumulative stress exposures, in addition to differences in stress appraisals or perceptions of events as upsetting across five life domains. Their findings point to more nuanced relationships between race, stress exposure, and mental health that implicate stress appraisals as an important mediator that has differential effects for anxiety versus depressive symptoms. This work adds to a growing body of research on race differences in pathways and contributors to mental health and the role of cultural psychosocial resources at individual, interpersonal, and community levels (i.e., religiosity, social support, racial identity) that buffer the effects of stress on health and mental health for Black Americans.

Qin and colleagues (2020) examined the role of social support (contact and perceived support) from extended family and friends in moderating the long-term effects of everyday discrimination on depressive symptoms among older African Americans (using Wave 8 through Wave 13 of HRS data). Findings confirm prior work indicating that more perceived discrimination is associated with more depressive symptoms over time. Consistent with the support mobilization model of stress and coping, reports of discrimination were positively associated with depressive symptoms over time for older adults who had more support from extended family and friends, but discrimination and support were unrelated for those who had lower family/friend support. The authors’ work confirms the impact of discrimination on depressive symptoms over time, as well as extended family and friend social support as a psychosocial resource that older African Americans employ to cope with everyday discrimination.

White and colleagues (2020) used data from the HRS to explore the association between discrimination trajectories and depressive symptoms among middle-aged and older Black adults. Repeated-measures latent profile analyses (a person-centered analysis approach that ascertains patterns of variables among persons that share similar attributes) were used to identify discrimination trajectories over time that reflected: (a) persistently high racial discrimination, (b) moderate discrimination, or (c) persistently low discrimination. Findings indicate that discrimination trajectories had different patterns of association with depressive symptoms; older age was associated with stronger relationships between discrimination and elevated depressive symptoms. This study of the association between perceived discrimination and depressive symptoms contributes to the literature by examining the cumulative patterning of exposure to discrimination over time and how that is associated with differences in risk and vulnerability to discrimination stress.

An abundance of research verifies the impact of loneliness on the physical and mental health of older adults. Taylor and Nguyen (2020) extend this work by examining the potential moderating effects of race in the relationship between loneliness and depressive symptoms. Using data on older Black and White adults from the HRS Core survey and Psychosocial Leave Behind Questionnaire, they examine an interaction term for race and loneliness within the context of relevant sociodemographic and psychosocial covariates. Their findings confirm the link between loneliness and depressive symptoms for both older Black and White adults. Further, although older Black adults have higher prevalence rates of both loneliness and depressive symptoms, the relationship between loneliness and depressive symptoms is stronger for Whites than Blacks. These findings complicate our understanding of how and why loneliness is associated with depressive symptoms and what accounts for the differential effect. Promising areas of future investigation include examining qualitative differences in how older Black and White adults experience loneliness. Loneliness interventions that capture racial and ethnic differences have the potential for developing more effective approaches for alleviating depression and depressive symptoms among older Black and White adults.

Gutierrez and colleagues (2020) address the little recognized but important issue of depression among older Mexican adults (60 years and older) and the association between time-use activities and depressive symptoms. Using 2012 and 2015 waves of data from the Mexican Health and Aging Study, they explore associations between baseline time use (2012) with depressive symptomatology in 2015. Their analysis identifies sociodemographic characteristics associated with elevated depressive symptoms in 2015. In addition, specific time-use domains (e.g., hobbies, volunteering) were associated with lower odds for depressive symptoms for both women and men. Their study adds to research indicating that engagement in activities and social support are important protective resources that buffer the negative influence of social isolation on mental health outcomes.

Using data from the 2010 and 2012 HRS, Thorpe and colleagues (2020) extend prior research on high allostatic load and compromised physical health. They focus on associations between allostatic load and mental health outcomes in examining chronic stress and depressive symptoms (Center for Epidemiological Studies-Depression [CES-D] eight-item scale) among Black men aged 50–101 years. Their findings that Black men who report higher allostatic load (assessed by biomarker values) were more likely to report having three or more depressive symptoms adds to the body of work indicating that physiological adaptation to chronic stress is detrimental to both physical and mental health.

Johnson Nicholson and colleagues (2020) explore the association between both distal (childhood material resources and life events) and current material and psychosocial resources and experiences and mental health outcomes among older African American adults from the Georgia Centenarian Study. Their work clarifies the association between present-day material and psychosocial resources and mental health (depressive symptoms). Further, their findings for the impact of childhood financial well-being indicate that this factor is not mediated through current life circumstances. Instead, evidence indicates that childhood financial well-being has a direct positive influence on self-rated mental health at older ages, emphasizing the importance of early life factors on later status. The study underscores the complex associations and pathways existing between early life experiences, current material and psychosocial resources, and mental health among older African American adults.

Finally, Mitchell and colleagues’ (2020) study of hopelessness represents a new and fruitful area of inquiry for studies of discrimination among older Black adults. Black adults have been noted for their higher rates of hopefulness relative to more materially advantaged racial groups (i.e., Whites) and in the face of dire life circumstances. Conversely, hopelessness (i.e., sense of despair) is a risk factor for poor mental and physical outcomes and all-cause mortality. In recent years, hopelessness has come to characterize the life outlook and mental and behavioral health patterns of Whites who are middle-aged, rural, and noncollege-educated (i.e., deaths of despair). Mitchell and colleagues examine whether psychosocial resources (social support and religious involvement) buffer the impact of self-reported everyday discrimination on hopelessness within a nationally representative sample of middle-aged and older Black adults from the HRS. Their work expands our understanding of the pathways through which psychosocial resources operate in buffering the impact of discrimination on hopelessness. Importantly, their work explores within-group and age-specific differences in how these relationships are manifested. 

In summary, the articles in this special issue contribute to a much needed and growing body of research on the mental health of older racial and ethnic minority adults. The articles address several psychosocial issues in relation to mental health status including loneliness and hopelessness, risk factors for poor mental health such as obesity and stress, as well as protective factors such as religion and social support. In total, this collection contributes five state-of-the-art literature reviews and 10 empirical studies to the small body of literature on the mental health of older adults from racial and ethnic minority groups. As an initial attempt to examine mental health among racial and ethnic minority populations, this special issue focuses on Black, Latinx, and Asian Americans older adults. Going forward, it is my hope that continuing research on mental health among older adults will focus on other racial and ethnic minority groups that remain understudied such as American Indians and Alaskan Natives, Native Hawaiian and Pacific Islanders, and Middle-Eastern and North African older adults. Research efforts that reflect both between- and within-group variability in the life histories and circumstances of diverse groups will be of immeasurable benefit in enriching our understanding of the diversity of aging lives and experiences.
